# Quiet Stance Postural Control in Women Who Have a History of Brain Injury from Intimate Partner Violence: A Preliminary Study

**DOI:** 10.1089/neur.2025.0015

**Published:** 2025-05-28

**Authors:** Bradi R. Lorenz, Shambhu P. Adhikari, Jonathan D. Smirl, Colin Wallace, Quinn Malone, Brian H. Dalton, Paul van Donkelaar

**Affiliations:** ^1^ Faculty of Health and Social Development, University of British Columbia, Kelowna, British Columbia, Canada.; ^2^ Faculty of Kinesiology, University of Calgary, Calgary, Alberta, Canada.; ^3^ Department of Kinesiology, Okanagan College, Kelowna, British Columbia, Canada.

**Keywords:** balance impairments, mental health, post-traumatic stress disorder, standing balance, sensorimotor function, women’s health

## Abstract

Intimate partner violence (IPV) frequently results in brain injury (IPV-BI) among survivors, with potential long-term effects for both physical and psychological health. This study aimed to examine the impact of chronic IPV-BI on postural control with (eyes open, [EO]) and without (eyes closed, [EC]) visual cues. We hypothesized that more exposure to a history of IPV-BI would be associated with greater postural control disruptions. During quiet stance, a force plate recorded forces and moments from which center of pressure (COP) variables were calculated to assess postural control. In addition, we sought to explore the relationship between psychological factors with assessments including indices of post-traumatic stress disorder (PTSD) (Clinician-Administered PTSD Scale), depression (Beck’s Depression Inventory), and anxiety (Beck’s Anxiety Inventory). Forty women survivors of IPV between the ages of 20 and 50 years participated, with the extent of exposure to IPV-BI measured using the Brain Injury Severity Assessment (BISA) tool on a scale of 0–8. Mediolateral (ML) COP displacement amplitude and variability, as well as anteroposterior (AP) COP velocity, was greater with EC than EO (*p* < 0.05). When participants were stratified into those with a low (0–2) and high (6–8) BISA score, participants in the high BISA (6–8) group exhibited greater COP area, ML COP amplitude and variability than those in the low BISA group (0–2; *p* < 0.05). Multiple linear regression analysis revealed that, independent of BISA score, PTSD symptoms contributed to changes in balance variables during the EO condition (*p* < 0.05). Taken together, our findings indicate the extent of exposure to a previous history of IPV-BI is linked to impairments in postural control as assessed by a variety of COP parameters. Given that standing balance is critical for function and mobility during activities of daily living, postural control assessments could serve as a valuable tool in diagnosing chronic IPV-BI. Thus, our study emphasizes the need for further research to better understand the physiological and psychological factors related to IPV-BI.

## Introduction

Intimate partner violence (IPV) is a pattern of physical violence, sexual violence, stalking, and/or psychological harm with coercive control.^1,2^ In Canada, 44% of women who had ever been in an intimate partner relationship have reported experiencing some kind of physical, psychological, or sexual violence in their lifetime.^3^ According to the Centers for Disease Control, approximately one-quarter of women experience severe physical violence by an intimate partner during their lifetime, such as being slammed against a wall, hit, strangled, or beaten.^4^ These violent events often include injuries to the head, face, and neck, with up to 92% of women recruited from women’s shelters or emergency rooms reporting being hit in the head or face by their partner.^5^ As such, the physical violence related to IPV can also result in brain injury (IPV-BI). IPV-BI is highly prevalent, with studies showing 35–88% of women who have experienced IPV are likely suffering a BI, and more than half of IPV survivors are experiencing multiple BIs.^6–11^

Despite the high incidence rate of IPV-BI, there has been relatively little quantitative analyses on the associations between the severity or frequency of BI and its resulting pathophysiological and psychopathological sequelae.^12^ Most of the studies conducted on IPV-BI examined survivors whose BIs were remote in time,^6,13–15^ with a limited number of studies focusing on survivors 1–2 weeks post injury.^16,17^ Exposure to IPV-BI is also typically long-term in nature, with women often being exposed to repeated episodes of violence, going through numerous attempts before permanently leaving an abuser, and are at the highest risk of being murdered when attempting to leave or report the abuse.^14,18^ The pathophysiological consequences of IPV-BI likely includes tissue deformation and direct damage to vascular, neuronal, and glial structures due to biomechanical forces.^19^ In addition, women who have experienced IPV may be subjected to non-fatal strangulation (NFS) that can result in BIs through the pathological effects of hypoxia and/or ischemia.^6^ These mechanisms can result in chronic negative mental and physical health outcomes, including the development of post-traumatic stress disorder (PTSD), depression, anxiety, suicidality, and decreased cognitive function.^6,20–22^

In addition to these aforementioned negative mental, physiological, and physical health outcomes, IPV-BI may also involve changes to balance and postural control. From a biomechanical perspective, maintenance of upright posture during static or quiet stance requires that the vertical projection from the whole-body center-of-mass (COM) must fall within the limits of the base of support.^23^ To accomplish this, adjustments in the underfoot center of pressure (COP) are used to guide the COM towards equilibrium. Larger and/or faster movements of the COP (and by extension the COM) have typically been viewed as a worsening of postural control.^23^ Disruptions to postural stability have been shown immediately following sport-related concussion and are thought to persist at least 3–5 days post-injury,^24–27^ with some studies showing balance disturbances up to one month or more following injury.^28^ Another study looking at chronic balance deficits in military veterans with a history of mild traumatic brain injury (mTBI) exhibited a variety of deficits in balance control along with heterogeneous sensory integration problems, even with their most recent mTBI being years prior.^29^

As more than half of IPV survivors experience multiple BIs, it is important to look at the results of studies examining changes in postural control in participants with a history of multiple BIs, whether that be from sports or military injuries.^6,29^ While there is an established dose-response relationship between the number of concussions and risk of suffering a repeat concussion,^24,30–32^ research on cumulative deficits in postural control following multiple concussions has yielded mixed results.^23,26^ Wasserman and colleagues found that athletes with multiple concussions showed a greater proportional loss of balance acutely than athletes who experienced their first concussion.^33^ However, another study observed that fully-recovered participants with a history of three or more concussions did not exhibit any differences in quiet stance COP parameters compared with individuals who had never sustained a concussion.^23^

In addition to IPV-BI, survivors also experience many psychopathological comorbidities, including PTSD, anxiety, and depression,^34^ which may contribute to balance deficiencies.^35–39^ For example, a systematic review,^39^ reported that depression was associated with impaired gait and posture. Anxiety may also be related to balance dysfunction; whereas anxiety-inducing situations can modify gait and balance performance in healthy individuals.^35–37^ Individuals with chronic postural instability may also develop state anxiety related to the anticipation of an actual or non-actual threatening event, such as a fall.^37^ In addition, military service members with persistent post-concussive symptoms after mTBI, and who developed PTSD, exhibited worse scores on affective measures that were associated with poor postural control, gait, and otolith function.^38^

The purposes of this study were to characterize different aspects of postural control to determine the effect of IPV-BI on quiet stance. It was hypothesized that quiet stance postural control would be impaired with greater exposure to IPV-BI, observed as greater COP area, and larger COP displacement amplitude, variability, and velocity. This study also sought to examine correlations between COP variables and psychopathological factors such as depression, anxiety, and PTSD in individuals with IPV-BI. It was hypothesized that greater IPV-BI exposure would be correlated with greater balance impairments, and this worsened balance performance would be associated with higher levels of depression, anxiety, and PTSD symptoms.

## Materials and Methods

### Participants

The principal criterion for study inclusion was at least one reported incident of IPV, however, participants were not excluded if they had experienced BI outside of the context of IPV. As such, 40 female participants between the ages of 20 and 50 years were recruited from local community partner sites in and around Kelowna, British Columbia, Canada (demographic information can be found in Table 1). All aspects of the study were described to the women prior to written informed consent being obtained from participants, with all questions about the study being explained prior to data collection. The study was approved by the clinical research ethics board at the University of British Columbia (H16-02792).

**Table 1.** Demographic and Clinical Characteristics of the Female Participants Who Experienced IPV and were Recruited from Local Community Partner Sites in and Around Kelowna, British Columbia, Canada

Characteristics	Mean ± SD or number (%)			
	Full sample (*N* = 40)	Participants with BISA = 0 to 2 (*n* = 15)	Participants with BISA = 3 to 5 (*n* = 17)	Participants with BISA = 6 to 8 (*n* = 8)
Age (Years)	37.38 ± 7.37	37.53 ± 7.65	36.41 ± 6.66	39.13 ± 8.84
Education (Years)	13.50 ± 2.09	13.17 ± 1.48	14.09 ± 2.00	12.88 ± 3.04
Ethnicity				
Caucasian	24 (60.00)	11 (73.30)	7 (41.20)	6 (75.00)
Indigenous	7 (17.50)	2 (13.30)	5 (29.40)	0
Black	2 (5.00)	0	2 (11.80)	0
Immigrants	2 (5.00)	1 (6.70)	1 (5.90)	0
Not disclosed	5 (12.50)	1 (6.70)	2 (11.80)	2 (25.00)
BISA-frequency	2.28 ± 1.3	1.07 ± 0.59	2.65 ± 1.12	3.75 ± 0.46
BISA-recency	1.03 ± 1.25	0.07 ± 0.26	1.24 ± 1.25	2.38 ± 0.92
BISA-severity	0.35 ± 0.48	0.27 ± 0.46	0.35 ± 0.49	0.50 ± 0.54
BISA-total score	3.65 ± 2.11	1.40 ± 0.74	4.24 ± 0.75	6.63 ± 0.74
BDI	24.08 ± 12.95	22.07 ± 14.42	22.41 ± 10.97	31.38 ± 12.95
BAI	23.88 ± 13.25	21.87 ± 13.79	21.47 ± 12.87	32.75 ± 10.31
CAPS-4	117.30 ± 54.44	98.80 ± 54.17	125.88 ± 47.25	133.75 ± 65.70

Note: BAI, Beck’s Anxiety Inventory (0–7: minimal, 8–15: mild, 16–25: moderate, 26–63: severe); BDI, Beck’s Depression Inventory (0–9: minimal, 10–18: mild, 19–29: moderate, 30–63: severe); CAPS-4, Clinician-Administered PTSD Scale-4 (∼75% of participants reported very severe PTSD symptoms); PTSD, post-traumatic stress disorder; BISA, Brain Injury Severity Assessment Tool (0–2: low, 3–5: medium, 6–8: high); N, Full sample; n, sub-sample, SD, standard deviation.

### Experimental procedure

Data in this study was collected as part of a larger study conducted at the University of British Columbia’s Okanagan campus. All participants were tested over two sessions separated by three to seven days. The protocol for each session with the corresponding assessments is described in prior publications.^11,12,34^ The assessments included indices of PTSD (Clinician-Administered PTSD Scale 4 [CAPS-4])^40^ depression (Beck’s Depression Inventory [BDI])^41^ anxiety (Beck’s Anxiety Inventory [BAI])^42^ and the Brain Injury Severity Assessment (BISA).^6^ The second session consisted of an assessment of current symptom burden and laboratory assessments of cerebrovascular, sensorimotor, neurocognitive, and blood biomarker measures.^11,12,34^ The current paper focuses on sensorimotor function. All women were familiarized with the testing procedures prior to participation and did not exercise or consume caffeine/alcohol 12 hours prior to testing.^43^

IPV-BI was assessed using the BISA tool,^6,7,44^ a semi-structured interview used to characterize BI exposure resulting from impacts targeting the head, face, and neck, and NFS that resulted in alterations of consciousness and/or signs or symptoms consistent with BI during episodes of IPV. The BISA yields an overall score of 0–8 composed of recency, frequency, and severity subcomponents—with lower scores (0–2) representing little to no previous exposure to IPV-BI and higher scores (6–8) representing significant exposure to IPV-BI.^6,7,44^ The recency subcomponent is defined as the number of weeks since the most recent BI from IPV occurred (0 = >52 weeks, 1 = 27–52 weeks, 2 = 14–26 weeks, and 3 = 0–13 weeks ago). For the purpose of this study, BI occurring ≥6 months prior was considered chronic.^45^ The frequency subcomponent is related to the number of IPV episodes resulting in probable BI (1 = 1–5 BIs, 2 = 6–10 BIs, 3 = 11–15 BIs, and 4 = ≥16 BIs). Finally, the severity score reflects whether any IPV episodes resulted in loss of consciousness or post-traumatic amnesia (1) or not (0). The BISA scores have been shown to be associated with disruptions to neurocognitive function, white matter integrity, and functional connectivity.^6,7,44^ In the current article the BISA scores were binned into low (0–2), moderate (3–5), and high (6–8) groups.

**FIG. 1.**
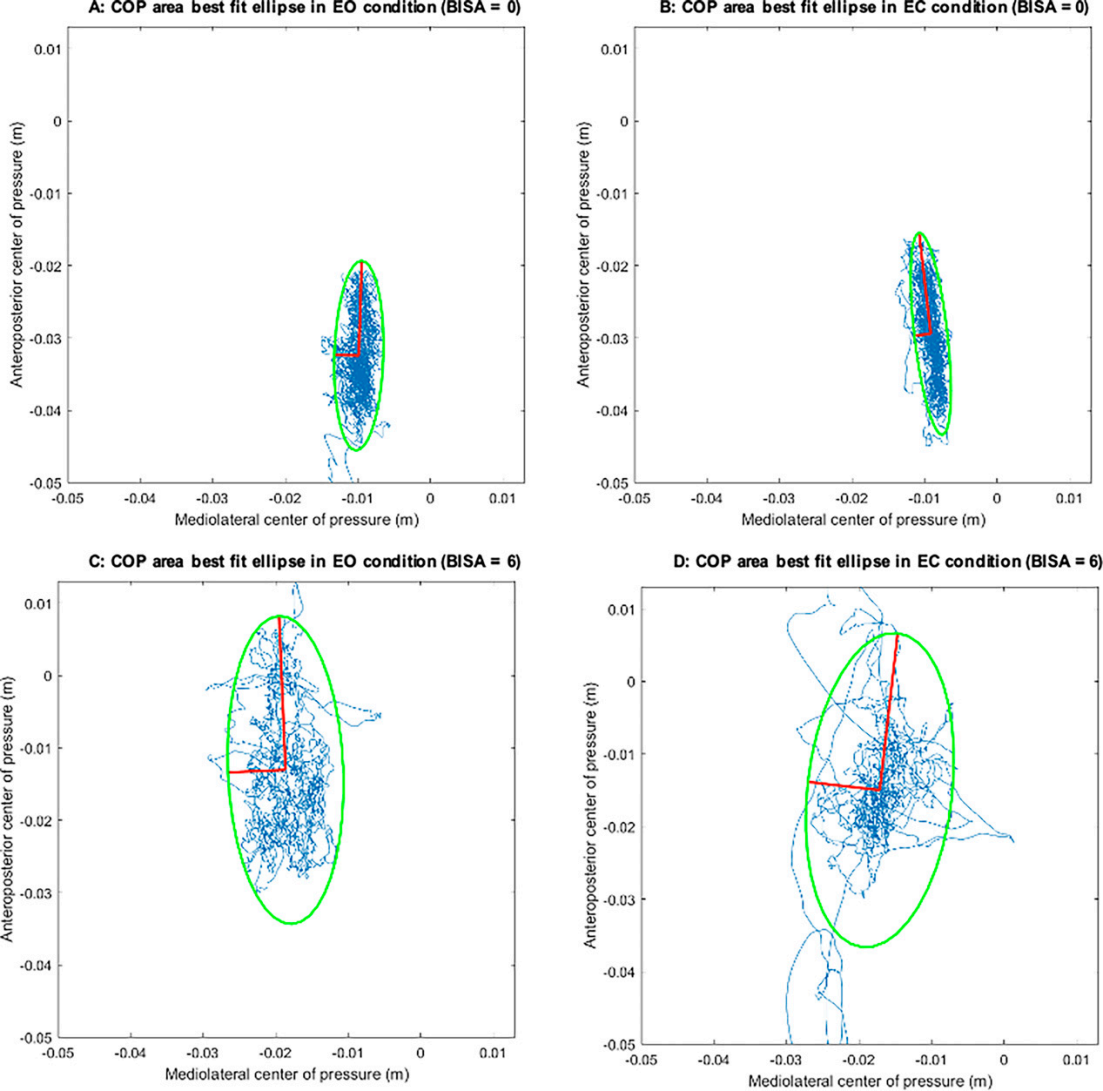
Sample trace of balance control representing best fit ellipse of COP Area (indicated in green) for two participants, one from the low BISA (0–2) and one from the high BISA (6–8) group. The best-fit ellipse represents the central tendency and dispersion of the COP area and provides a visual representation of postural stability, with its size and orientation reflecting the extent and direction of COP variability in the anteroposterior and mediolateral directions. **A:** Eyes open, BISA = 0. **B:** Eyes closed, BISA = 0. **C:** Eyes open, BISA = 6. **D:** Eyes closed, BISA = 6. COP, center of pressure; BISA, Brain Injury Severity Assessment.

Participants completed one 60-second quiet-stance trial for each of two conditions (eyes open [EO] and eyes closed [EC]). Although the trial duration may influence the outcome dependent variables,^46^ we are confident that any impact of trial length on quiet stance performance is controlled owing to similar durations across conditions. Before each trial, participants were given the following instructions: *“Stand quietly on the force plate with your hands on your hips and your feet hip width apart and do not move your feet unless it is necessary to maintain your balance.”* In addition, for the EO trial, participants were instructed to *“keep your gaze focused on the wall.”* Stance width was not normalized or measured; rather, we relied on participants to self-select a stance width that approximated their hip width and was comfortable for them. Ground reaction forces and moments were recorded by the force plate (True Impulse, Northern Digital Inc., Waterloo, ON) in the mediolateral (ML), anteroposterior (AP), and vertical directions relative to the center of the plate and sampled at 1000 Hz. Forces and moments were then filtered digitally with a zero lag, 4th-order, low-pass digital Butterworth filter using a 10 Hz cut-off frequency. COP profiles were calculated, and postural control was assessed using COP amplitude, variability, and velocity in the AP and ML directions as well as total COP area (Fig. 1).^23^ COP amplitude was defined as the difference between the maximum and minimum recorded COP samples along an axis during a trial. COP variability was defined as the standard deviation of COP displacement along an axis. COP velocity was defined as the mean absolute difference between each consecutive COP sample divided by the inter-sample interval (1 ms). The COP area was defined as the area circumscribed by a best-fit ellipse. A custom-written MATLAB script (version 23.2.0, R2023b, The MathWorks Inc., Natick, Massachusetts, USA) was used to reduce the collected data to the reported outcome measures.

### Statistical analyses

Descriptive statistics (mean ± SD) were conducted to analyze the demographic and clinical characteristics of the cohort. A one-way ANOVA was used to determine if there were any differences between the three groups based on the BISA score (low BISA = 0–2 vs. medium BISA = 3–5 vs. high BISA = 6–8). A two-way mixed ANOVA was used to explore a potential three group ([low BISA], [medium BISA], [high BISA]) × two conditions ([EO], [EC]) interaction, within-participant effects (EO vs. EC), and between-participant effects (low vs. medium vs. high BISA score). *Post-hoc* multiple comparisons were conducted with the Bonferroni correction method. SPSS offers Bonferroni-adjusted significance tests for pairwise comparisons. This adjustment is available as an option for *post-hoc* tests and for the estimated marginal means feature.^47^ Multiple linear regressions were conducted to evaluate the relationships between various balance control variables (COP area, ML and AP COP amplitude, variability, and velocity) with BISA score, and different covariates including age, ethnicity, education, BDI, BAI, and CAPS-4. The partial Eta squared (η^2^_p_) was calculated to determine whether the effects were small (0.01), medium (0.06), or large (0.14).^48,49^ Significance was set at *p* < 0.05. All statistical analyses were performed using SPSS Statistics version 29.0 (IBM Corp, Armonk, NY).

## Results

The mean values for age, education, BDI, BAI, and CAPS-4 were not different between the three BISA groups (Table 1, *p* > 0.05). When considering the entire sample (*n* = 40), only two participants scored zero on the BISA, indicating that 95% of participants experienced signs and symptoms consistent with a BI. Further, the BISA total, frequency, and recency scores were greatest for the high BISA score group (*p* < 0.05), whereas the severity score was not different between groups (Table 1, *p* = 0.556). The mean BISA recency score for the entire sample was 1.03 ± 1.25, indicating that the average time since the most recent BI for this sample was between 27 and 52 weeks. Participants reporting the most recent BI experienced their injury between 0 and 13 weeks before assessment (Table 1). Greater than half of the participants in the current study (*n* = 21) had a BI recency score of 0, indicating that their most recent BI was over a year prior. In addition, 67.5% (*n* = 27) of participants indicated that their most recent BI occurred six or more months previously and therefore are considered to have chronic BI.

**FIG. 2.**
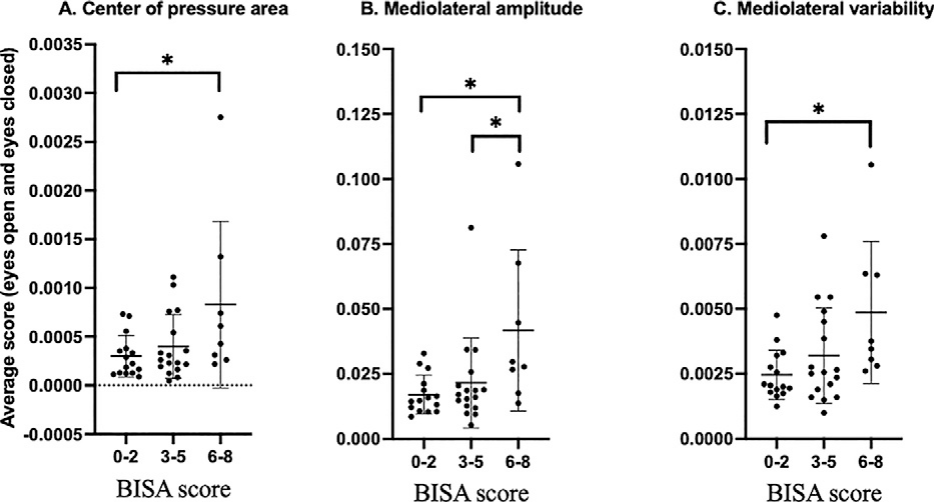
Post-hoc comparison following two-way mixed ANOVA on various balance control variables: **(A)** Center of pressure area, **(B)** Mediolateral amplitude, and **(C)** Mediolateral variability. The average score (eyes open and eyes closed) is presented in the Y-Axis. Three different BISA load groups (0–2, 3–5, and 6–8) are shown in the X-Axis. The Bonferroni method was used as a *post-hoc* test to adjust *p* values for multiple comparisons using SPSS. The *p* value (‘significance’) obtained here is adjusted so that it can be compared directly to 0.05. BISA, Brain Injury Severity Assessment.

Significant differences were found between COP area (η^2^_p_ = 0.12), ML-Amp (η^2^_p_ = 0.21), and ML-Var (η^2^_p_ = 0.20) among participants across the three BISA groups (low vs. medium vs. high BISA score). Bonferroni corrected *post-hoc* tests were conducted for the three BISA groups for COP Area, and ML COP amplitude and ML COP variability, which revealed greater COP area (*p* = 0.031) and ML COP variability (*p* = 0.012) for the high BISA score group compared to the low (Fig. 2). Also, the high BISA score group exhibited a greater ML COP amplitude than low (*p* = 0.011) and medium (*p* = 0.041) groups (Fig. 2). There was no BISA score group (low, medium, and high) × vision (EO, EC) interaction for any COP variable (Table 2). Further, a main effect for vision was detected for ML COP amplitude (*p* = 0.023) as well as AP COP variability (*p* = 0.046) and velocity (*p* = 0.019), indicating these scores were higher with EC than EO (Table 2).

**Table 2.** Two-Way Mixed ANOVA Looking at Postural Control Variability Between Eyes Open and Eyes Closed Conditions Across Various Brain Injury Scores (BISA: 0–2 vs. 3–5 vs. 6–8) (N = 40)

Variables	Low BISA (0–2) (*n* = 15)		Medium BISA (3–5) (*n* = 17)		High BISA (6–8) (*n* = 8)		Two-way mixed ANOVA					
	Mean ± SD											
	EO	EC	EO	EC	EO	EC	F*	Groups X conditions	F**	Within-subjects	F*	Between-subjects
COP Area (m^2^)	0.00031 ± 0.00026	0.00030 ± 0.00022	0.00040 ± 0.00044	0.00040 ± 0.00036	0.00081 ± 0.00080	0.00085 ± 0.0012	0.02	0.977	0.02	0.888	3.82	0.031*
ML-Amp (m)	0.019 ± 0.009	0.016 ± 0.007	0.257 ± 0.031	0.017 ± 0.009	0.057 ± 0.062	0.027 ± 0.015	1.65	0.206	5.61	0.023*	5.02	0.012*
AP-Amp (m)	0.036 ± 0.019	0.038 ± 0.011	0.034 ± 0.016	0.039 ± 0.015	0.049 ± 0.023	0.049 ± 0.020	0.24	0.788	0.90	0.349	2.35	0.109
ML-Var	0.003 ± 0.001	0.002 ± 0.003	0.003 ± 0.002	0.003 ± 0.002	0.005 ± 0.003	0.005 ± 0.004	0.01	0.985	0.94	0.338	4.69	0.015*
AP-Var	0.006 ± 0.003	0.006 ± 0.002	0.005 ± 0.002	0.006 ± 0.003	0.006 ± 0.003	0.008 ± 0.003	0.20	0.818	4.26	0.046*	1.65	0.206
ML-Vel (m/s)	0.012 ± 0.002	0.011 ± 0.002	0.012 ± 0.005	0.011 ± 0.004	0.015 ± 0.006	0.014 ± 0.016	0.02	0.984	0.47	0.496	1.26	0.297
AP-Vel (m/s)	0.014 ± 0.002	0.016 ± 0.004	0.014 ± 0.004	0.017 ± 0.005	0.019 ± 0.008	0.026 ± 0.026	0.96	0.393	6.04	0.019*	2.39	0.106

Note: BISA, Brain Injury Severity Assessment Tool (0–2: low, 3–5: medium, 6–8: high); COP, center-of-pressure; ML, mediolateral; AP, anteroposterior; Amp, amplitude; Var, variability; Vel: velocity; EO, eyes open; EC, eyes closed.

Significant at *p* < 0.05, *df = 2.37; **df = 1.37. Bold text indicates significance set at *p* < 0.05.

Multiple linear regression analyses were conducted to explore the relationship between the different COP variables and BISA score with age, education, ethnicity, BAI, BDI, and CAPS-4 as covariates. As displayed in Table 3, BISA score was related to COP area (*p* = 0.004), AP COP velocity (*p* = 0.018), as well as ML COP amplitude (*p* = 0.002), variability (*p* = 0.005) and velocity (*p* = 0.024) with EO; whereas with EC, BISA score was related to COP area (*p* = 0.037), AP COP velocity (*p* = 0.049), and ML COP amplitude (*p* = 0.024) and ML COP variability (*p* = 0.035). The CAPS-4 scores demonstrated an association with COP area (*p* = 0.006), as well as ML COP amplitude (*p* = 0.002) and ML COP variability (*p* = 0.004) with EO, but not with EC. Finally, age was associated with ML COP amplitude (*p* = 0.030) with EO. All other variables did not significantly contribute to the relationship with the various balance control variables (Table 3).

**Table 3.** Multiple Linear Regression Analysis to Demonstrate the Relationships of Various Balance Control Variables with BISA Score, and Different Covariates (Age, Education, Ethnicity, BAI, BDI, and CAPS) in a Sub-Sample of Women Who Experienced IPV (*n* = 34)

Covariates	COP area (m^2^)		ML-Amp (m)		AP-Amp (m)		ML-Var		AP-Var		ML-Vel (m/s)		AP-Vel (m/s)			
	Std. ß	*p*	Std. ß	*p*	Std. ß	*p*	Std. ß	*p*	Std. ß	*p*	Std. ß	*p*	Std. ß	*p*		
Eyes open																
BISA (Score)	0.463	**0.004** ^*^	0.477	**0.002** ^*^	0.320	0.076	0.456	**0.005** ^*^	0.238	0.202		0.398		**0.024** ^*^	0.404	**0.018** ^*^
Age (Y)	0.278	0.063	0.315	**0.030** ^*^	0.154	0.908	0.216	0.153	0.097	0.592		0.192		0.247	0.137	0.391
Education (Y)	0.074	0.617	0.003	0.981	−0.014	0.936	0.075	0.620	0.032	0.862		−0.030		0.856	−0.247	0.133
Ethnicity	0.090	0.612	0.174	0.308	−0.112	0.596	0.139	0.446	−0.165	0.456		0.023		0.910	−0.053	0.784
BAI (Score)	0.158	0.517	0.245	0.295	−0.072	0.802	0.290	0.248	−0.140	0.643		0.155		0.569	0.040	0.880
BDI (Score)	0.387	0.099	0.334	0.135	0.310	0.258	0.392	0.102	0.259	0.366		−0.064		0.807	−0.265	0.295
CAPS-4 (Score)	−0.822	**0.006** ^*^	−0.902	**0.002** ^*^	−0.466	0.168	−0.891	**0.004** ^*^	−0.327	0.351		−0.501		0.122	−0.205	0.505
Eyes closed																
BISA (Score)	0.387	**0.037** ^*^	0.419	**0.024** ^*^	0.258	0.175	0.399	**0.035** ^*^	0.278	0.134	0.148		0.451	0.356	**0.049** ^*^	
Age (Y)	0.259	0.146	0.088	0.615	−0.017	0.925	0.199	0.266	0.264	0.147	0.026		0.893	0.247	0.166	
Education (Y)	−0.032	0.859	−0.127	0.473	−0.198	0.291	−0.028	0.878	−0.041	0.820	0.057		0.768	−0.008	0.963	
Ethnicity	−0.050	0.817	−0.120	0.574	−0.071	0.750	−0.058	0.788	−0.021	0.922	−0.233		0.322	−0.118	0.585	
BAI (Score)	−0.074	0.802	−0.147	0.615	0.101	0.741	−0.178	0.549	0.066	0.826	−0.273		0.396	−0.100	0.733	
BDI (Score)	0.116	0.674	0.159	0.563	0.111	0.703	0.118	0.675	0.159	0.574	−0.213		0.481	−0.202	0.467	
CAPS-4 (Score)	−0.151	0.655	−0.015	0.965	−0.209	0.556	−0.003	0.994	−0.352	0.311	0.410		0.271	−0.004	0.991	

Std. ß, Standardized Beta; BAI, Beck’s Anxiety Inventory (0–7: minimal, 8–15: mild, 16–25: moderate, 26–63: severe); BDI, Beck’s Depression Inventory (0–9: minimal, 10–18: mild, 19–29: moderate, 30–63: severe); CAPS-4, Clinician-Administered PTSD Scale-4 (∼75% of participants reported very severe PTSD symptoms); PTSD, post-traumatic stress disorder; BISA, Brain Injury Severity Assessment Tool (0–2: low, 3–5: medium, and 6–8: high).

COP, center-of-pressure, ML, mediolateral; AP, anteroposterior; Amp, amplitude; Var, variability: Vel, velocity.

^*^ Significant at *p* < 0.05.; IPV, Intimate partner violence.

## Discussion

The current study provides one of the first examinations of balance control in women who have experienced chronic IPV-BI. The main findings are three-fold. First, the balance control variables COP area, AP velocity, ML COP amplitude, and ML COP variability are modulated by the amount of exposure to IPV-BI. The greater the BISA score, the greater the disruption to these balance control measures. Second, PTSD symptoms contributed to balance control variations in IPV-BI survivors, particularly with EO. Third, differences in balance control variables were observed during EO versus EC conditions.

While the negative effects of acute BI from other injury mechanisms on postural stability are well documented, existing literature shows mixed results when it comes to examining the effects of chronic BI. One study of former high school football players found that chronic participants with a history of concussion (at least two concussions, at least 15 years prior) presented with more regular balance performance in the ML direction while balancing double-legged with EC on a sway-referenced support surface.^50^ Despite their history of concussion, trained athletes may exhibit better balance control compared to their non-active counterparts.^51^ Despite this, the authors,^50^ suggested the loss-of-complexity hypothesis of aging and disease best supported their findings. This theory implies that the complex integration of visual, vestibular, and somatosensory processing into sensorimotor responses is impaired by concussion, which manifests as less intricate postural control.^50,52^ However, many other previous studies have reported that individuals with acute concussion and those with a history of concussion present with balance impairments that persist beyond the typical 7–10 day recovery window.^26,53–57^ This is consistent with our results, which found differences in COP Area as well as ML COP amplitude and ML COP variability across the three BISA score groups regardless of visual condition (Table 2). Due to the chronic and repetitive nature of IPV, this population is comparable to those with a history of concussions and repetitive sub-concussive head impacts in other populations such as contact sport athletes, military personnel, and people experiencing homelessness.^10,29,58–61^ The results of the current study show postural control is worsened—marked by greater COP parameters (Table 2, Fig. 1, Fig. 2)—in the high BISA score group compared with the low. In addition, our results indicate that the BISA score is the best at predicting changes in many of the balance variables in both the EO and EC conditions compared to the other examined factors. While the BISA is a self-reported screening tool and therefore does not provide a diagnosis of BI, a number of studies have successfully employed it to gain insight into the impacts of BI in IPV.^11,12,34,62–64^ In addition, analogous screening tools used in populations with suspected BI from other injury mechanisms have been successfully used to examine the impact on various aspects of brain function.^6,65^ Further, only two participants in this study reported “0” or no possible BI on the BISA questionnaire. While this study could have benefited from comparing a “no BI” group to the high (6–8) BI group, this was not possible given the sample population. Future studies should include participants before IPV manifests/escalates to the point of physical incidents leading to potential BI. Regardless, overall, the results of the current study reveal the clear impact of chronic IPV-BI on postural control (Table 2).

A novel finding of the current study was that PTSD symptoms—assessed via the CAPS-4 tool—contributed to changes in balance measures in the EO condition. In particular, with greater PTSD symptomology, the variables of COP area, ML COP amplitude, and ML COP variability were decreased in the EO condition. However, similar changes were not apparent with EC. While this is a novel finding, it may be consistent with previous work that found eye closure resulted in increased body sway for PTSD patients.^66^ The authors of this paper suggested that PTSD patients, with their EC are unable to scan the environment for threatening cues, and therefore, the noted increase in body sway could be an indicator of elevated fight-or-flight behavior in response to eye closure.^66^ Informed by those previous results, it can be speculated that the reduced body sway in the EO condition could be due to the ability of the participants to scan their environment for the threatening cues. Another explanation is that this reduction in body sway may also be related to tonic immobility, a state of physical immobility associated with extreme stress and the development of PTSD.^66^ Therefore, the observed reduction of postural sway with EO could also be attributed to protective, cautionary, or fear-related behaviours. Furthermore, a study investigating the effects of mTBI and PTSD on blast-exposed military veterans found that persons with a history of PTSD had reduced postural stability relative to those without PTSD, independent of the effects of mTBI.^67^ Individuals with military-relevant mTBI exhibit a variety of chronic balance deficits involving heterogeneous sensory integration problems that may be related to PTSD, mTBI, or a combination of both.^29,67^ The results of the current study show that PTSD symptomology contributed to changes in the balance variables independent of BISA score. This may be related to proprioception and/or vestibular feedback integration becoming impaired by a process that overlaps with the pathophysiological processes of PTSD. Those with higher PTSD symptom scores may rely more on vision for feedback on their movements, which is suggestive for other movement impairments to exist for tasks that do not rely on vision for some or all of their performance.^68^ Further research is required to more thoroughly examine the interactions between PTSD, BI, and balance control, particularly as they relate to proprioception and vestibular feedback.

While both depression and anxiety scores—measured by the BDI and BAI, respectively—did not contribute to changes in balance measures in this study, evidence from past studies in non-IPV populations indicated that anxiety and/or depression do disrupt postural control and gait and may also be linked to mobility dysfunction.^35,39^ On average, the participants in this study fell on the moderate-high levels of both the BDI and BAI. Thus, the scores landed on a narrow range, and this may explain why differences in balance control were not modulated by depression and anxiety in these participants.^6,11,20^ It is crucial that future research explores the relationship between mental health conditions and postural control in this population. Investigating how anxiety and depression may interact with the long-term effects of IPV-BI could lead to comprehensive treatment strategies that address both psychological and physical impairments in this population.

The results of the current study are consistent with previous studies that have reported worsening balance control in EC versus EO conditions.^23,69–72^ These results have been consistent over a multitude of populations, including those with sports concussion and Alzheimer’s disease.^23,69^ The EC condition is described as the more challenging condition as it largely taps into proprioceptive and vestibular functions due to the withdrawal of visual feedback, where deficits in these sensory systems may play a substantive role in balance impairment.^73^ The EC condition can thus be considered more challenging because it places a greater demand on these alternative sensory modalities, and any dysfunction or compensatory issues within these systems—such as those following concussion or mTBI—could exacerbate balance problems.^74^ This suggests individuals with impaired proprioceptive or vestibular function, such as those with a history of IPV-BI, may be particularly vulnerable to balance deficits under EC conditions. Of note, one participant in this study, who scored an 8 on the BISA, demonstrated extreme differences in the EO versus EC conditions. In the EO condition, the participant was wobbly but could maintain balance. However, in the EC condition, the participant could not maintain any aspect of postural control. Therefore, this participant was excluded from the study as they could not complete the balance task. This participant provides a case example of extreme proprioceptive and vestibular changes and vulnerability to balance deficits with EC and a high BISA score.

### Implications, limitations, and future directions

This study provides evidence that balance assessments could be useful in the diagnosis of chronic, remote BI in survivors of IPV. By assessing balance control, clinicians may be able to identify subtle yet significant impairments linked to chronic, remote IPV-BI. This approach could help address gaps in the current diagnostic process, as survivors of IPV often face challenges in receiving appropriate medical attention for any injury that occurred from past incidences of IPV. This injury can be BI but also includes injuries caused by physical assaults and impacts anywhere else on the body, as well as NFS. Incorporating balance assessments into routine evaluations could enhance the accuracy of diagnoses, leading to targeted interventions (for example, physical therapy) and improved outcomes for IPV survivors living with the long-term effects of BI.

This study is not without limitations. First, the sample size is relatively small, and a control group was not included, which may limit our ability to detect subtle, yet potentially significant effects. Second, participant recruitment was restricted to a few key community organizations that support women who have experienced IPV. As a result, the generalizability of our findings to the broader IPV population may be limited, as the participants recruited for this study had often been exposed to IPV at remote instances in time and had since been removed from their violent situation. Moreover, the shelter these participants were recruited from was a high-barrier shelter that precluded substance use, so those currently using substances were not included in the current study. This study also did not include recruitment from hospitals or clinics. Therefore, participants who did not make use of the specific community organizations would not have been recruited. In addition, within the population of IPV survivors, help-seeking behavior and experiences within the healthcare system are affected by societal perceptions and stigma of IPV, normalization of violence, and individual characteristics such as race, socioeconomic status, education, insurance status, and disability status.^75^ Third, there was considerable variation in the time since the participants’ most recent incidence of IPV, which could introduce a large variability in the balance control measurements. However, balance deficits in military populations have been reported to persist 9+ years post-injury, so there is broader literature highlighting these chronic deficits in balance control and may actually strengthen the generalizability of our findings to survivors of IPV with remote injuries.^29^ In addition, due to the cross-sectional design of the study and the presence of multiple comorbid conditions in participants, we are unable to establish causal relationships between IPV-BI and the observed effects on balance control.

## Conclusions

The current study found that postural control is linked to the amount of exposure to IPV-BI as marked by greater COP area and displacement during quiet stance. In addition, PTSD symptoms were observed to contribute to balance while participants had their EO, this may be related to tonic immobility, a state of physical immobility associated with extreme stress and the development of PTSD. These results underscore the complexity of the relationship between IPV-BI and postural control, highlighting the need for further research to elucidate the underlying mechanisms as well as to identify other potential factors—both physiological and psychological—that may impact postural control in survivors of IPV-BI.

## Transparency, Rigor, and Reproducibility Summary

This is a primary study of prospectively collected data in Kelowna, British Columbia, Canada. The materials and methods are described in detail in articles previously published.^11,12,23^ Participant selection, settings, measurements, and procedures in those articles are described in a simpler and clearer form to enhance the reproducibility of the studies. Women survivors of IPV who met eligibility criteria were recruited at each site. Women between the ages of 18 and 67 years who had experienced at least one instance of IPV in the past were recruited from various women-serving organizations in the community. The data of 40 women with complete datasets were broken down into severity of IPV-BI as determined by the BISA score.

Descriptive statistics (mean ± SD) were conducted to analyze the demographic and clinical characteristics of the cohort. Analysis of variance was used to discover if there were any differences between the groups based on the BISA scores, as well as to explore group*condition interaction, within-subjects effects, and between-subjects effects. *Post-hoc* multiple comparisons were conducted with a Bonferroni correction factor. Multiple linear regression was conducted for evaluation of the relationships of various balance control variables with BISA score, and different covariates such as age, ethnicity, education, depression, anxiety, and PTSD symptoms. Significance was set at *p* < 0.05.

Approval to conduct this study was received from the University of British Columbia clinical research ethics board, and all participants provided written informed consent prior to participation in this study. Data is securely stored by the principal investigator.

## Abbreviations Used

AP: anteroposterior
BAI: Beck's Anxiety Inventory
BDI: Beck's Depression Inventory
BISA: Brain Injury Severity Assessment
CAPS: Clinician-Administered PTSD Scale
COM: center-of-mass
COP: center of pressure
EC: eyes closed
EO: eyes open
IPV: intimate partner violence
IPV-BI: intimate partner violence caused brain injury
ML: mediolateral
mTBI: mild traumatic brain injury
PTSD: post-traumatic stress disorder
